# Case Report: Toxic encephalopathy caused by repeated inhalation of liquid sealant

**DOI:** 10.3389/fpubh.2022.920310

**Published:** 2022-08-05

**Authors:** Zixin Wen, Ping Dai, Zhiqiang Zhou, Lanlan Guo, Tongyue Zhang, Aerbusili Genjiafu, Tianzi Jian, Yaqian Li, Baotian Kan, Xiangdong Jian

**Affiliations:** ^1^School of Nursing and Rehabilitation, Cheeloo College of Medicine, Shandong University, Jinan, China; ^2^Department of Poisoning and Occupational Diseases, Emergency Medicine, Qilu Hospital of Shandong University, Cheeloo College of Medicine, Shandong University, Jinan, China; ^3^School of Public Health, Cheeloo College of Medicine, Shandong University, Jinan, China; ^4^Department of Geriatric Medicine, Qilu Hospital, Cheeloo College of Medicine, Shandong University, Jinan, China

**Keywords:** toxic encephalopathy, organic solvent, liquid sealant, inhalant addiction, poisoning

## Abstract

Short-term exposure to high levels of organic solvents, as well as long-term exposure to small doses, can damage the central nervous system, thereby leading to toxic encephalopathy. However, toxic encephalopathy caused by long-term inhalation of liquid sealant is rarely reported. This study describes the clinical data of a case of toxic encephalopathy caused by repeated inhalation of liquid sealants and discusses the pathophysiological characteristics and treatment of organic solvent toxic encephalopathy. This report aims to strengthen the understanding of this disease among clinical staff.

## Introduction

Now, the effects of inhaling substances and their impact on the society and users' health have become a major issue for researchers and government. Meanwhile, the abuse of other kinds of volatile substances, such as glue and nail varnish remover, has also been reported (1). Most of these volatile substances are mainly organic solvents such as toluene and n-hexane (2). Due to their volatile and lipophilic properties, organic solvents can easily enter the human body through skin and mucous membrane contact, as well as by air inhalation. The lipid-rich nervous system is especially susceptible to the effects of organic solvents, which can cause toxic encephalopathy by affecting neurotransmitter function and damaging the white matter. Acute poisoning usually manifests as headache, dizziness, fatigue, alterations in consciousness, and other non-specific neurological dysfunctions. The early symptoms of chronic-onset toxic encephalopathy are insidious; cognitive impairment, asthma, and personality changes may occur in the later stages (3–5). On August 19, 2021, our department admitted a patient with toxic encephalopathy caused by the repeated inhalation of liquid sealant fumes, which is a relatively rare occurrence. This case report may serve as a reference for the diagnosis and treatment of similar patients.

## Case description

A 34-year-old man was admitted to the Department of Poisoning and Occupational Diseases, Qilu Hospital of Shandong University, on August 19, 2021. He reported sudden development of headache, dizziness, nausea, and vomiting 3 days earlier. These symptoms were followed by limb weakness, slurred speech, and slow reaction time. Upon experiencing intermittent convulsions and subsequent loss of consciousness, his family members immediately transported him to the local hospital.

Chest, abdominal, and pelvic computed tomography, as well as cerebral venous magnetic resonance angiography, showed no abnormalities. Brain magnetic resonance imaging (MRI) showed patchy and slightly longer signal shadows in the bilateral cerebral hemispheres, subcortical region, outer capsule, and thalamus. Fluid-attenuated inversion recovery (FLAIR) and diffusion-weighted imaging (DWI) showed a high signal intensity and involvement of the bilateral parietal lobes, thus suggesting toxic encephalopathy (Supplementary Figure 1).

**Figure 1:**
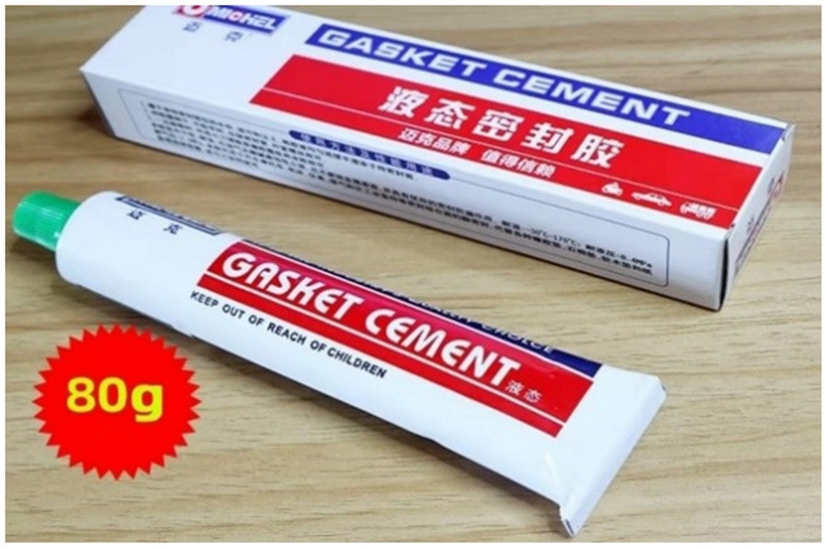
The liquid sealant (nitrile type) inhaled by the patient.

The local hospital administered nutritional nerve and symptomatic supportive treatment. As few improvements were observed after 2 days of treatment, the patient was transferred to our hospital for further care. According to the patient's family members, the patient had first contacted the liquid sealant (nitrile type) at work 10 years earlier, and he felt that the smell was very good. After that, he still had a similar feeling when he contacted it again. Six months earlier, he had been exposed to the substance again and he became addicted. He then started buying the liquid sealant online and inhaling it at home (Figure 1). The frequency of inhalation had increased in the previous 6 months: to ~10 times/day, for ~15 min every time. He had had a total of 32 inhalations the previous month. However, when family members and patients were asked about the patient's medical history in the early days of admission, they denied any history of exposure to special substances. Therefore, we did not collect patients' blood for toxicological analysis during the initial period of admission.

## Diagnostic assessment

The following parameters were recorded upon physical examination on admission: temperature (36.3°C), pulse rate (70 beats/min), respiration rate (16 breaths/min), and blood pressure (105/58 mmHg). The patient's general condition was poor; he was conscious but had slow reactions and difficulty talking. Yellow staining was not observed on the skin or mucous membranes, and there was no swelling of the superficial lymph nodes. The pupils were large and round bilaterally, with a diameter of ~3 mm, and sensitive to light reflection. Neither cyanosis of the lips nor pharyngeal congestion was detected. No abnormal masses were palpated in the neck. Both sides of the chest were symmetrical, and the range of motion was equal. The breath sounds of both lungs were clear, and no rales were heard. The heart rhythm was steady, and no pathological murmur was heard in the valve auscultation area. The abdomen was flat and soft, and none of the following features were observed: intestinal type, peristaltic waves, tenderness, rebound pain in the whole abdomen, abnormal bowel sounds, and touch under the liver, spleen, and ribs. No spinal or limb deformities were observed; the muscle strength of both the upper and lower limbs was categorized as grade 3. Physiological reflexes were intact, and no pathological reflexes could be elicited. These findings led to a diagnosis of toxic encephalopathy due to organic solvents.

The following laboratory parameters were documented after hospital admission: red blood cell (RBC): 5.16 × 10^12^/L, white blood cell (WBC): 5.84 × 10^9^/L, and platelets: 267 × 10^9^/L counts; and hemoglobin (HGB) level: 84 g/L. The coagulation series, liver function, renal function, as well as electrolyte, myocardial enzyme, and blood glucose levels were not significantly abnormal (Table 1). The electrocardiogram (ECG) was normal. On admission, we immediately administered comprehensive treatment that included oxygen inhalation, ECG monitoring, blood oxygen saturation monitoring, glucocorticoids (0.9% normal saline 100 ml + betamethasone 10.52 mg *via* intravenous drip once daily), sodium valproate, vitamin B1, mecobalamin, nalmefene, injection of neurotrophic factors (rat nerve growth factor), and other symptomatic supportive therapies.

**Table 1:** Results of the laboratory examinations of the patient.

**Admission time**	**Day 1**	**Day 3**	**Day 7**	**Day 21**	**Day 51**	**Reference value**
WBC (10^9^/L)	5.84	6.92	8.03	7.29	4.98	3.5–9.5
NEU (%)	76.90	82.10	71.60	61.3	59.3	40–75
RBC (10^12^/L)	5.16	4.75	4.69	4.60	5.24	4.3–5.8
HGB (g/L)	84.0	80.0	78.0	79.0	90.0	130–175
MCV (fL)	60.7	63.8	64.2	66.5	65.3	82–100
HCT (%)	31.30	30.30	30.10	30.60	34.20	40.0–50.0
PLT (10^9^/L)	267	276	291	250	258	125–350
ALT (IU/L)	20	10	49	76	18	9–50
AST (IU/L)	21	9	23	20	17	15–40
TBIL (mmol/L)	9	4.3	3.6	5.9	6.1	5.0–21.0
BUN (mmol/L)	3.4	6.80	7.30	5.30	4.70	2.30–7.80
Cr (μmol/L)	49	57	54	56	61	62–115
Glu (mmol/L)	7.0	7.73	4.34	4.55	—	3.90–6.10

*WBC, white blood cells; NEU, neutrophils; RBC, red blood cells; HGB, hemoglobin; MCV, mean corpuscular volume; HCT, hematocrit; PLT, platelet; ALT, alanine aminotransferase; AST, aspartate aminotransferase; TBIL, total bilirubin; BUN, urea nitrogen; Cr, serum creatinine; Glu, blood glucose*.

**Figure 2:**
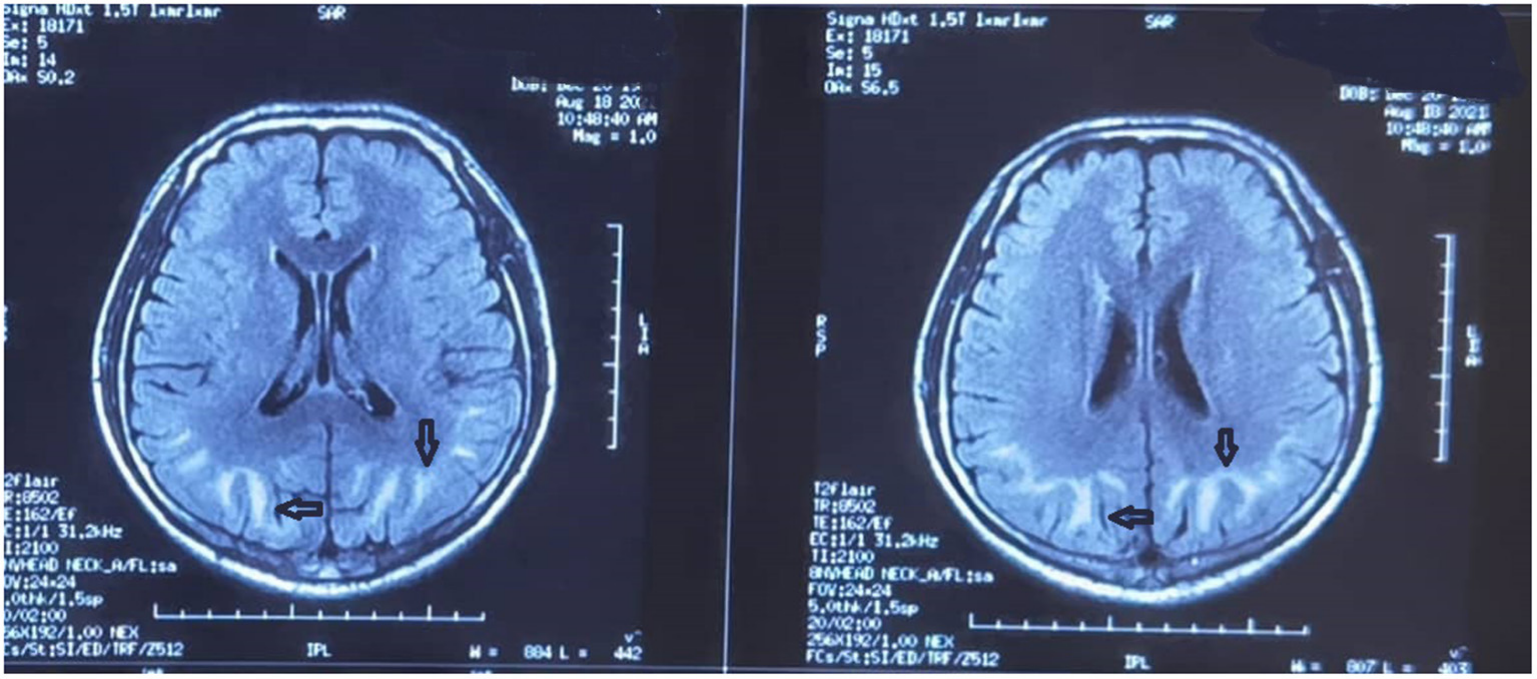
Brain magnetic resonance image obtained on the 3rd day after admission. Strip-like long T1 and T2 signal shadows in the subcortical white matter of both cerebral hemispheres are evident. There is a high intensity on both T2-fluid-attenuated inversion recovery and diffusion-weighted imaging. A fuzzy demarcation is visible between the skin and medulla. The septum pellucidum is widened and the midline structures are centered.

No significant changes were observed in the patient's condition by the 3rd day after admission. The RBC count and HGB level were 4.75 × 10^12^/L and 80 g/L, respectively. Cerebral T1 and T2 MRI showed long strip-like signal shadows in the subcortical white matter of both cerebral hemispheres, with hyperintensity on T2-FLAIR and hyperintensity on DWI (Figure 2). The demarcation between the cortex and medulla was blurred. The septum pellucidum was widened and the midline structures were centered.

**Figure 3:**
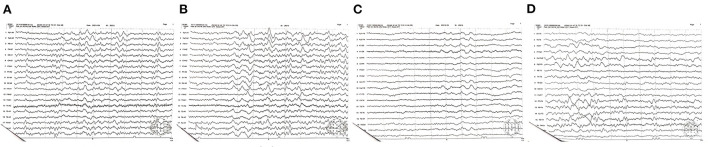
**(A–D)** Electroencephalogram obtained on the 6th day after admission. A high degree of abnormality and a poor background are evident. Low and medium amplitude activity θ dominates, while the medium amplitude δ rhythm is intermittent.

On the 6th day after admission, the electroencephalogram showed a high degree of abnormality (poor background, low and medium amplitude θ activity, and intermittent medium δ rhythm; Figure 3). Therefore, we continued to administer glucocorticoids, nutritional nerve therapy, anti-infection, liver and stomach protective therapy, parenteral nutrition, and other comprehensive treatments. The patient's vital signs were stable on the 7th day; while no convulsions were observed, the patient remained unresponsive. The RBC count, WBC count, and HGB level were 4.69 × 10^12^/L, 8.03 × 10^9^/L, and 78.0 g/L, respectively. Other laboratory tests showed no obvious abnormalities. Betamethasone infusion was switched to oral prednisone (60 mg/day), which was subsequently maintained at a dose of 30 mg/day (one tablet). The patient's condition was stabilized, and no further convulsions were observed. The patient was transferred back to the local hospital for functional rehabilitation exercises on August 27, 2021; oral treatment with mecobalamin was maintained, while prednisone administration was gradually discontinued.

The patient returned to our hospital for re-examination on the 12th day after discharge. His condition was stable, and his symptoms were slightly relieved; however, his reaction time was still slow and his speech was not fluent. No significant changes in blood laboratory parameters were observed. MRI showed symmetrical and lace-like long T1 signals and long T2 signals in the dentate nucleus, basal ganglia, and white matter of the cerebellum (Supplementary Figure 2). Both FLAIR and DWI showed high signals, while the apparent diffusion coefficient yielded a low signal. Brain fluid cavity system morphology and its signal were normal, and the midline structures remained in the middle. The electroencephalogram obtained on the 14th day after discharge showed mild abnormalities, slow background activity, as well as diffuse, slightly low, and medium amplitude θ activity (Supplementary Figure 3). Electromyography showed no obvious neuromuscular abnormalities (Supplementary Figure 4).

An additional review was conducted on the 42nd day after discharge. The symptoms had slightly improved, and the blood laboratory parameters were as follows: RBC count: 5.24 × 10^12^/L, HGB level: 90.0 g/L, and WBC count: 4.98 × 10^9^/L. Laboratory tests of electrolyte levels and liver and kidney function showed no obvious abnormalities. Brain MRI showed that the long T1 and T2 signals were symmetric and patchy in the white matter, basal ganglia, thalamus, and dentate nucleus of the cerebellum in both hemispheres (Supplementary Figure 5). FLAIR showed a slightly high signal, while DWI showed a high signal. No abnormalities were observed in the brain fluid cavity, and the midline structures were in the middle. The electroencephalogram showed no obvious abnormalities (Supplementary Figure 6). The patient continued to perform rehabilitation exercises at home.

## Discussion

Inhalant abuse is a significant public health problem in many countries. Acute poisoning with volatile substances usually follows the deliberate inhalation of vapor to intoxication. This phenomenon is known as “glue sniffing,” inhalant abuse, solvent abuse or volatile substance abuse (2). Glue, shoe polish, toluene, and lighter fluid are commonly abused inhalants. Acute effects of inhalant intoxication include dizziness, ataxia, salivation, flushing, motor retardation, blurred vision, nystagmus, and can lead to sudden death in severe cases (6). Long-term effects include myocardial damage, pulmonary toxicity, hepatic toxicity, and cognitive and memory problems. Toxic encephalopathy is rarely reported after inhaling glue (7). In this study, after excessive inhalation of liquid sealant, the patient presented with several symptomse, including headache, dizziness, nausea and vomiting, limb weakness, and convulsions. In addition, MRI examination of the patient showed toxic encephalopathy.

Organic solvent toxic encephalopathy is a type of toxic leukoencephalopathy. While acute organic solvent toxic encephalopathy is primarily associated with headache, dizziness, nausea, vomiting, and fatigue, severe cases may involve blurred consciousness, seizures, coma, and even death. The onset of chronic toxic encephalopathy due to organic solvents is often insidious and is associated with non-specific symptoms such as dizziness, headache, and fatigue. With continual exposure to organic solvents, such symptoms may persist for at least 5–10 years, with symptom severity being positively correlated with exposure time. Thereafter, the patient may be at an increased risk of deterioration in their performance of regular daily activities, as well as a progression in memory decline and mood and sleep disorders (8, 9). Due to the lack of specific symptoms, the diagnosis of organic solvent toxic encephalopathy mainly depends on the following findings: (1) a history of long-term exposure to large amounts of organic solvents; (2) symptoms typical of nervous system injury, accompanied by objective findings on clinical examination; and (3) the exclusion of other organic brain diseases and primary mental diseases (10). Finally, combined with the patient's exposure history, clinical manifestations, and MRI findings, we confirmed the diagnosis of acute organic solvent toxic encephalopathy.

MRI, as an auxiliary clinical examination, plays an important role in the diagnosis of organic solvent toxic encephalopathy. In the present case, MRI showed strip-like long T1 and T2 signal shadows in the subcortical white matter of both cerebral hemispheres. Hyperintensity was observed with both T2-FLAIR and DWI, and a fuzzy demarcation was evident between the skin and the medulla. A previous study conducted among patients with chronic toxic encephalopathy reported the presence of cortical or cerebellar atrophy on MRI (11). However, in the present case, the patient exhibited diffuse cerebral edema with no cortical or cerebellar atrophy. This finding suggests that the patient may have inhaled large amounts of organic solvents within a short period of time in a confined space, leading to acute organic solvent toxic encephalopathy.

The management of organic solvent toxic encephalopathy comprises prevention and symptomatic treatment, as there is currently no specific treatment available. In the present case, we administered glucocorticoid shock therapy at an early stage to improve the intracranial edema, clinical symptoms, and overall prognosis, in accordance with previous reports (12). In addition, drugs were provided to promote brain blood circulation, brain material metabolism, and functional recovery. Sodium valproate was administered to stop the convulsions. In addition, it is necessary to emphasize the importance of optimizing nursing, counseling, and rehabilitation protocols, to lower the risk of complications and treat inhalant addiction. In the present case, after receiving active symptomatic treatment and ceasing the liquid sealant inhalation, the patient's clinical symptoms disappeared, brain swelling significantly improved, the lesion disappeared, and a clear boundary between the cortex and the medulla was re-established on MRI. The patient in this case did not experience any additional epileptic symptoms following treatment with sodium valproate and neurotrophic drugs.

## Conclusion

Inhalant abuse is a major public health problem. In China, volatile organic solvents are not listed as drugs for control, but the abuse of volatile organic solvents makes people addicted, causing harm to their bodies. Therefore, in the future, we should strengthen the monitoring of inhalant abuse, enhance people's awareness of inhalant abuse, and further improve the relevant laws and regulations.

## Data availability statement

The original contributions presented in the study are included in the article/Supplementary material, further inquiries can be directed to the corresponding authors.

## Ethics statement

The studies involving human participants were reviewed and approved by the Ethics Committee of the Qilu Hospital of Shandong University. The patients/participants provided their written informed consent to participate in this study. Written informed consent was obtained from the individual(s) for the publication of any potentially identifiable images or data included in this article.

## Author contributions

ZW and PD designed the study, participated in data analysis, and wrote the manuscript. ZZ, LG, TZ, AG, TJ, and YL designed the study, performed the majority of experiments, analyzed the data, and drafted the manuscript. BK and XJ participated in the revising of the manuscript. All authors contributed to the article and approved the submitted version.

## Conflict of interest

The authors declare that the research was conducted in the absence of any commercial or financial relationships that could be construed as a potential conflict of interest.

## Publisher's note

All claims expressed in this article are solely those of the authors and do not necessarily represent those of their affiliated organizations, or those of the publisher, the editors and the reviewers. Any product that may be evaluated in this article, or claim that may be made by its manufacturer, is not guaranteed or endorsed by the publisher.

## References

[1] Sah SK Neupane N Thaiba AP Shah S Sharma A. Prevalence of glue-sniffing among street children. Nurs Open. (2019) 7:206–11. 10.1002/nop2.380 31871704 PMC6917933

[2] Djurendic-Brenesel M Stojiljkovic G Pilija V. Fatal intoxication with toluene due to inhalation of glue. J Forensic Sci. (2016) 61:875–8. 10.1111/1556-4029.13019 27122437

[3] Dick FD. Solvent neurotoxicity. Occup Environ Med. (2006) 63:221–6. 10.1136/oem.2005.022400 16497867 PMC2078137

[4] Sit G Letellier N Iwatsubo Y. Occupational exposures to organic solvents and asthma symptoms in the CONSTANCES cohort. Int J Environ Res Public Health. (2021) 18:9258. 10.3390/ijerph18179258 34501848 PMC8431091

[5] Letellier N Choron G Artaud F. Association between occupational solvent exposure and cognitive performance in the French CONSTANCES study. Occup Environ Med. (2020) 77:223–30. 10.1136/oemed-2019-106132 32075885 PMC7079188

[6] Jayanth SH Hugar BS Praveen S Chandra YPG. Glue sniffing. Med Leg J. (2017) 85:38–42. 10.1177/0025817216671106 27694447

[7] Tuchscherer J Rehman H. Metabolic acidosis in toluene sniffing. CJEM. (2013) 15:249–52. 10.2310/8000.2013.130974 23778000

[8] Mi T Han C Wang Y. Acute toxic leukoencephalopathy in migrant workers exposed to organic solvents in construction materials. Occup Environ Med. (2013) 70:435–6. 10.1136/oemed-2012-101302 23390197 PMC3664387

[9] Filley CM Halliday W Kleinschmidt-DeMasters BK. The effects of toluene on the central nervous system. J Neuropathol Exp Neurol. (2004) 63:1–12. 10.1093/jnen/63.1.1 14748556

[10] van der Hoek JA Verberk MM Hageman G. Criteria for solvent-induced chronic toxic encephalopathy: a systematic review. Int Arch Occup Environ Health. (2000) 73:362–8. 10.1007/s004200000119 11007338

[11] Keski-Säntti P Mäntylä R Lamminen A Hyvärinen HK Sainio M. Magnetic resonance imaging in occupational chronic solvent encephalopathy. Int Arch Occup Environ Health. (2009) 82:595–602. 10.1007/s00420-008-0368-3 18936955

[12] Dang J Chen J Bi F Tian F. The clinical and pathological features of toxic encephalopathy caused by occupational 1,2-dichloroethane exposure. Medicine. (2019) 98:e15273. 10.1097/MD.0000000000015273 31027082 PMC6831337

