# Supplementary material for: Case Report: Toxic encephalopathy caused by repeated inhalation of liquid sealant
Source: Front Public Health. 2022 Aug 5;10:920310. doi: 10.3389/fpubh.2022.920310 (PMC9389144; doi:10.3389/fpubh.2022.920310)
Supplement: Supplementary file 1 [file Data_Sheet_1.docx]

***Supplementary Material***

1. **Supplementary Figures and Tables**

**1.1 Supplementary Figures**


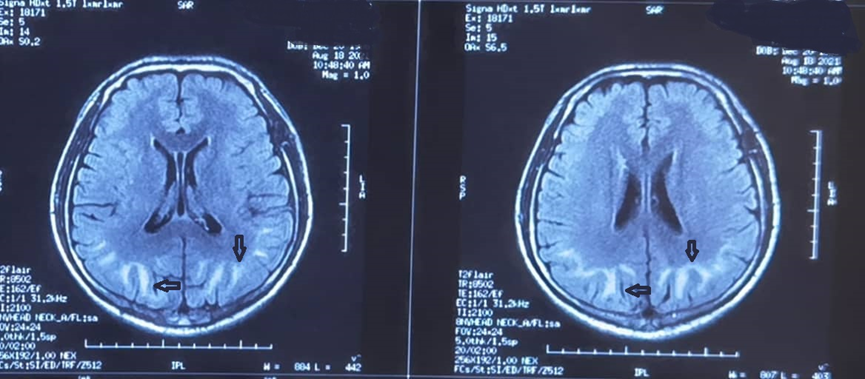


**Supplementary Figure 1.** Brain magnetic resonance image obtained at a local hospital on August 18, 2021, showing patellar, slightly longer T1 and T2 signal shadows in the bilateral cerebral hemispheres, including the subcortical region, outer capsule, and thalamus. Fluid-attenuated inversion recovery and diffusion-weighted images show a high signal intensity and the involvement of the bilateral parietal lobes, suggesting toxic encephalopathy.


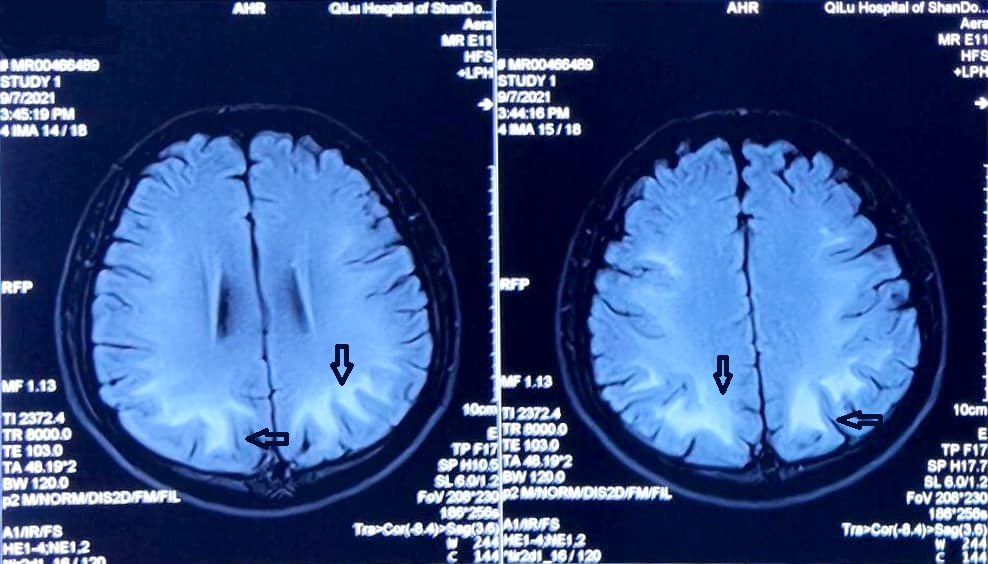


**Supplementary Figure 2.** Brain magnetic resonance image obtained on the 12th day after discharge. Symmetrical and patchy long T1 and T2 signals are evident in the cerebellar dentate nucleus, basal ganglia, and white matter of the cerebral hemisphere. Both fluid-attenuated inversion recovery and diffusion-weighted images show a high signal, while ADC shows a low signal. Brain fluid cavity system morphology and signal are normal, and the midline structure is centered.


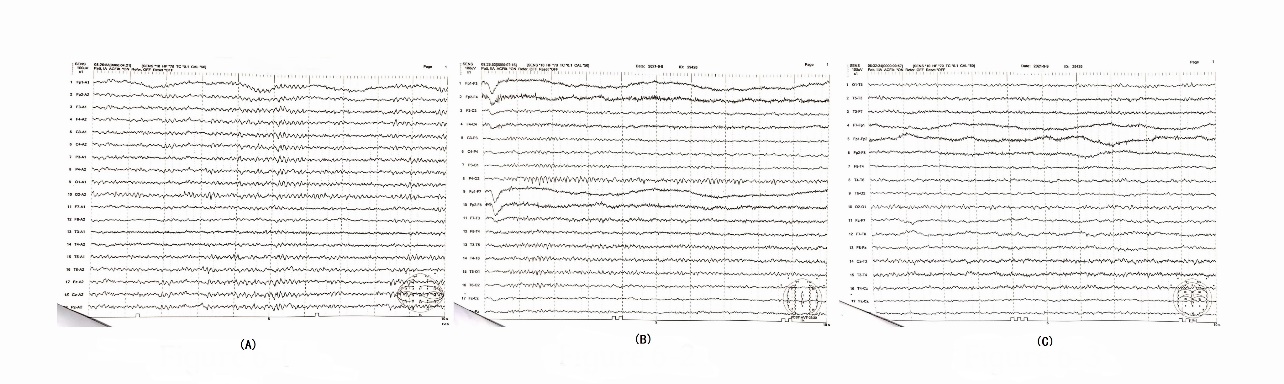


**Supplementary Figure 3.** Electroencephalogram obtained on the 14th day after discharge. Results show mild abnormalities, slow background activity, as well as diffuse, slightly low and medium amplitude θ activity.


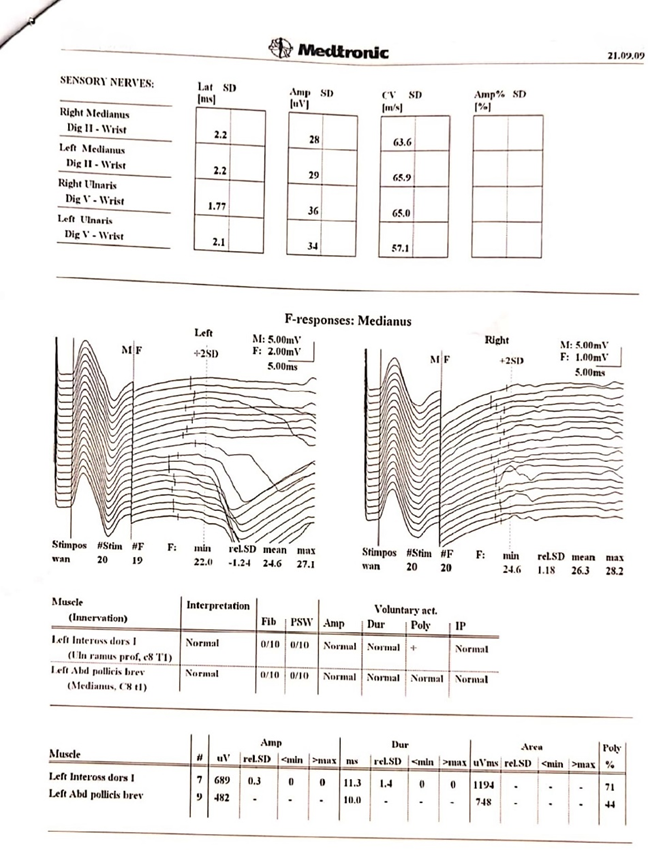


**Supplementary Figure 4.** Electromyogram obtained on the 14th day after discharge. No obvious neuromuscular abnormalities are apparent.


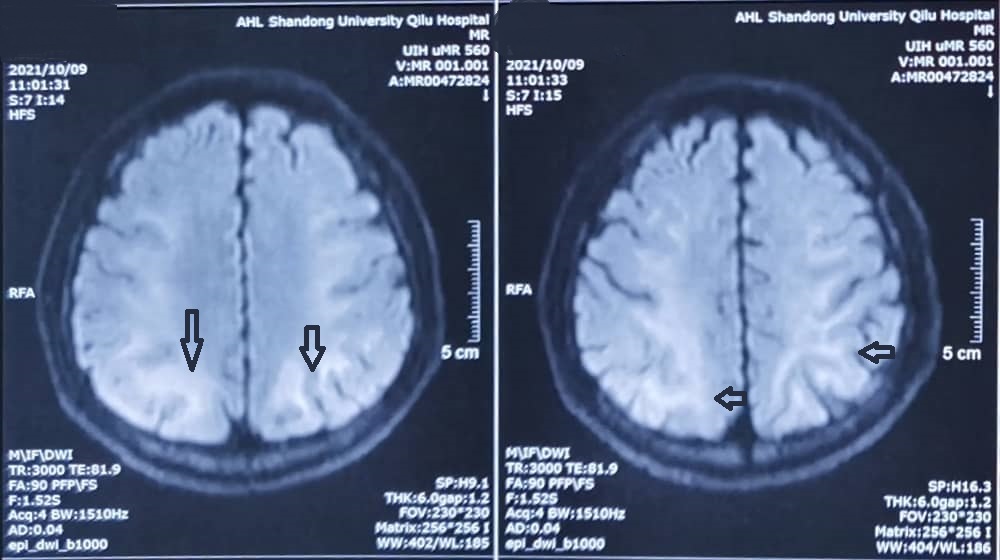


**Supplementary Figure 5.** Magnetic resonance images of the brain obtained 1 month after discharge. Symmetrical and patchy long T1 and T2 signals are evident in the white matter, basal ganglia, thalamus, and dentate nucleus of the cerebellum in both hemispheres. Fluid-attenuated inversion recovery image shows slightly high signals, while diffusion-weighted image shows high signals. No obvious abnormalities can be observed in the brain fluid cavity, and the median line structure is centered.


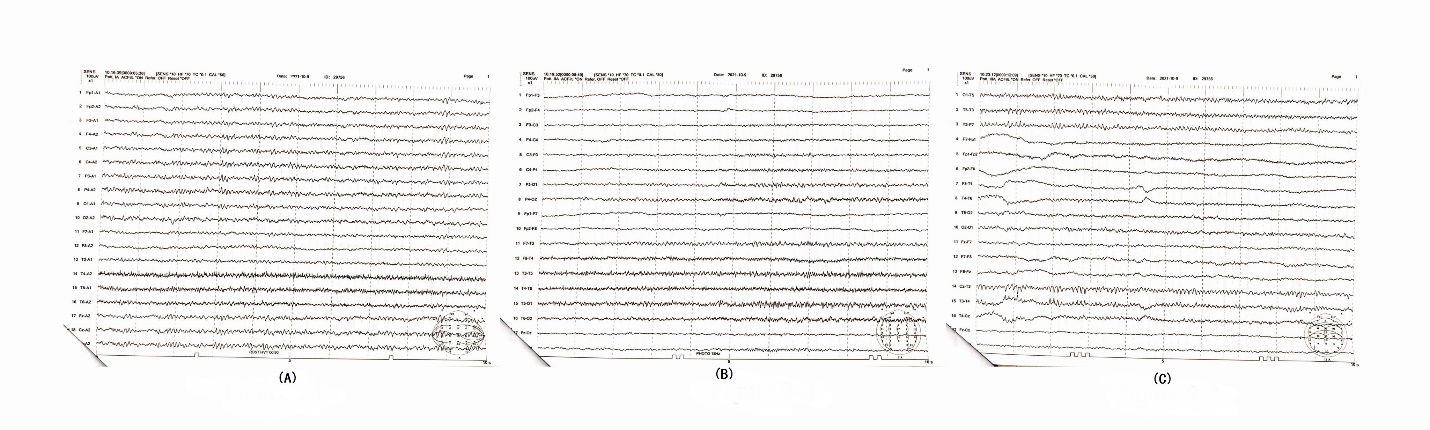


**Supplementary Figure 6.** Electroencephalogram obtained 1 month after discharge. No abnormalities are evident.
